# Genome-Wide Analysis of Gene Expression Provides New Insights into Cold Responses in *Thellungiella salsuginea*

**DOI:** 10.3389/fpls.2017.00713

**Published:** 2017-05-08

**Authors:** Jiangshan Wang, Quan Zhang, Feng Cui, Lei Hou, Shuzhen Zhao, Han Xia, Jingjing Qiu, Tingting Li, Ye Zhang, Xingjun Wang, Chuanzhi Zhao

**Affiliations:** ^1^Biotechnology Research Center, Shandong Academy of Agricultural Sciences, Shandong Provincial Key Laboratory of Crop Genetic Improvement, Ecology and PhysiologyJinan, China; ^2^Yantai Institute of China Agricultural UniversityYantai, China; ^3^College of Life Sciences, Shandong Normal UniversityJinan, China

**Keywords:** *Thellungiella salsuginea*, salt cress, cold stress, gene expression

## Abstract

Low temperature is one of the major environmental stresses that affects plant growth and development, and leads to decrease in crop yield and quality. *Thellungiella salsuginea* (salt cress) exhibits high tolerance to chilling, is an appropriate model to investigate the molecular mechanisms of cold tolerance. Here, we compared transcription changes in the roots and leaves of *T. salsuginea* under cold stress using RNA-seq. We identified 2,782 and 1,430 differentially expressed genes (DEGs) in leaves and roots upon cold treatment, respectively. The expression levels of some genes were validated by quantitative real-time-PCR (qRT-PCR). Among these DEGs, 159 (11.1%) genes in roots and 232 (8.3%) genes in leaves were annotated as various types of transcription factors. We found that five aquaporin genes (three *TIPs*, one *PIPs*, and one *NIPs)* responded to cold treatment. In addition, the expression of *COR47, ICE1, and CBF1* genes of *DREB1/CBF*-dependent cold signaling pathway genes altered in response to low temperature. KEGG pathway analysis indicated that these cold regulated genes were enriched in metabolism, photosynthesis, circadian rhythm, and transcriptional regulation. Our findings provided a complete picture of the regulatory network of cold stress response in *T. salsuginea*. These cold-responsive genes could be targeted for detail functional study and utilization in crop cold tolerance improvement.

## Introduction

Plants generally are rooted in one place, and have to face drought, salinity, high temperature, cold, and other adverse stresses which may cause significant loss of crop yield (Boyer, 1982; Kawasaki and Bohnert, 2001). Low temperature is one of the major environmental stresses that affect plant growth and development, crop yield and quality. In plant tissues, the intercellular fluid generally has a higher freezing point than the intracellular fluid. When temperature decreased below freezing point, intercellular spaces of plant tissues form ice prior to intracellular region. So, the water potential decreases rapidly outside the cells, and causes the movement of water from inside the cell to the intercellular spaces. Consequently, cold stress could lead to severe cellular dehydration (Thomashow, 1998). In addition, low temperature can lead to the formation of adhesion between the intercellular ice and the cell walls and membranes (Levitt, 1980). Low temperature damage could lead to growth inhibition, wilting, and weak seedling.

During the evolution history, most plants developed the capacity to tolerate cold. Cold acclimation is a strategy for the plants to acquire freezing tolerance by a prior exposure to low nonfreezing temperature (Guy, 1990). To adapt to low temperature environment, many physiological and molecular changes occur during cold acclimation (Thomashow, 1999). Exposure to low temperature, linolenic acid and membrane lipid unsaturation increased, and the plasma membrane H^+^-ATPase activity increased and these changes are essential for the plants to withstand low temperature (Shi et al., 2008). In addition, calcium-dependent protein kinase confers cold tolerance via the regulation of calcium channel in plasma membrane (Xiong et al., 2002; Komatsu et al., 2007). Early studies identified a number of genes in plants which were response to cold treatment, and these genes were known as cold regulated (*COR*) genes (Thomashow, 1999; Lee et al., 2005). For example, a total of 939 cold regulated genes were identified in *Arabidopsis thaliana* (Lee et al., 2005). The cold regulated genes were involved in a variety of functions such as metabolism, protein synthesis, signal transduction, transcription regulation, and hormone biosynthesis and signaling (Thomashow, 1999; Lee et al., 2005).

Among these *COR* genes, a family of transcription factor known as C-repeat/dehydration-responsive element-binding (*CBF*) was identified as the key factor to regulate response to cold stress in many plants (Gao et al., 2002; Xiong and Fei, 2006; Nakamura et al., 2011; Wisniewski et al., 2011). In *Arabidopsis*, three members of *CBFs* were identified, including *CBF1, CBF2*, and *CBF3* (also name as *DREB1b, DREB1c*, and *DREB1a*, respectively). Overexpression of *CBF1* induced *COR* genes and increased freeze tolerance of the transgenic plants (Jaglo-Ottosen et al., 1998; Yamaguchi-Shinozaki and Shinozaki, 2001). Deletion of all three *CBF* genes the transgenic plants are extremely sensitive to freezing after cold acclimation, suggesting that the three *CBF* genes together are essential for cold acclimation (Zhao et al., 2016). Recently, *ICE1* (inducer of *CBF* expression 1) was identified as an upstream transcription factor regulating the transcription of *CBF*, and its overexpression activated the expression of *CBF* regulon under cold condition and improved freeze tolerance of the transgenic plants (Chinnusamy et al., 2003). However, transcriptome profiling experiments showed that the number of CBF regulon gene accounts only 6.5 % of the total number of *COR* genes, suggesting that other transcription factors are also involved in the regulation of *COR* genes and the low-temperature regulatory network beyond the CBF pathway is complex and highly interconnected (Park et al., 2015). Recently, many other *COR* genes were identified from *CBF-*independent pathways. For example, *SCOF-1* encodes a cold-inducible zinc finger protein from soybean, and *Osmyb4* encodes a member of *MYB* transcription factor from rice, these genes also contributed to cold tolerance in plants (Kim et al., 2001; Vannini et al., 2004).

*Thellungiella salsuginea* also named as *T. halophile* or salt cress, is a close relative of *Arabidopsis*. Compare to *Arabidopsis, T. salsuginea* exhibits higher tolerance to cold, and it could complete its life cycles at 5°C, and could survive at extreme low temperature of –21°C after cold acclimation (Griffith et al., 2007). Thus, *Thellungiella* was proposed as an appropriate model to investigate the molecular mechanisms of plant adapted to cold stress (Griffith et al., 2007; Amtmann, 2009). To illustrate how *Thellungiella* adapts to low-temperature, cold regulated genes were identified from both the mRNA and protein levels. In a survey of 3,628 *Thellungiella* cDNAs, 76 cold induced transcripts including *COR47, ERD10*, and *COR15b* were identified using microarray methods (Wong et al., 2006). Northern blot analysis demonstrated that some cold response genes (*CBF1, COR15a*, and *COR47)* from *Arabidopsis* were also induced in *Thellungiella* (Griffith et al., 2007). Two-dimensional electrophoresis (2-DE) approach was used in *Thellungiella*, and found 66 protein spots were significantly affected by cold in *Thellungiella* rosette leaves (Gao et al., 2009). These studies provided useful clues for understanding the mechanism of cold tolerance in *Thellungiella*. However, due to the limited genomic sequences, these studies fail to provide a comprehensive interpretation of the transcriptomic changes of *Thellungiella* in response to cold. To gain insight into the molecular networks underlying *Thellungiella* cold tolerance, more comprehensive genome-wide gene expression profiling studies are required.

Recently, the whole genome sequence of *Thellungiella* was completed, which provides new opportunity to understand the cold tolerance mechanism in *Thellungiella* (Dassanayake et al., 2011; Wu et al., 2012). In this study, we carried out genome-wide analysis of gene expression in roots and leaves of *Thellungiella* under cold treatment using RNA-seq technology. The aim of the study is to identify cold responsive genes and biological pathways that may contribute to cold tolerance in *Thellungiella*. We identified thousands of cold-responsive genes and provided an overall picture of the regulatory network in response to cold stress in *Thellungiella*. These cold-responsive genes could be targeted as potential candidates for further functional validation, and have potential application value for increasing cold tolerance in crops.

## Results and Discussion

### High Throughput Sequencing and Gene Expression Profiles

To gain the profiles of gene expression in *Thellungiella* under cold condition, eight cDNA libraries were constructed using roots and leaves under normal (control) and low temperature (cold) for 24 h, respectively. The cDNA libraries were sequenced by Illumina Hiseq2000 platform using the paired end method. After removing low quality, N-containing and adaptor-contaminated reads, a total of 96,305,447 clean reads were generated, with an average of ∼12 million reads per libraries (**Table 1**). Approximately 90% reads from leaves and 85% reads from roots were mapped to the *Thellungiella* reference genome, and about 7% reads from leaves and 3.5% reads from roots were mapped to multiple regions, respectively (**Table 1**). The RNA-seq raw sequencing data from this study have been submitted in the SRA database^1^ under BioProjects: PRJNA377594 (SRA: SRP101369).

**Table 1:** Summary of read numbers from leaves and roots under cold treatment.

Samples	Reads in leaf (%)				Reads in root (%)			
	Control 1	Control 2	Cold 1	Cold 2	Control 1	Control 2	Cold 1	Cold 2
Total clean reads	11,839,336	12,464,847	12,606,715	11,696,922	12,135,076	11,814,795	11,896,522	11,851,234
Mapped reads	10,682,216 (90.23%)	11,286,644 (90.55%)	11,306,780 (89.69%)	10,432,315 (89.19%)	10,299,132 (84.87%)	10,110,549 (85.58%)	10,284,125 (86.45%)	10,185,035 (85.94%)
Unique match	9,750,932 (82.36%)	10,387,629 (83.34%)	10,457,647 (82.95%)	9,659,258 (82.58%)	9,852,257 (81.19%)	9,659,737 (81.76%)	9,859,595 (82.88%)	9,775,273 (82.48%)
Multi-position Match	931,284 (7.87%)	899,015 (7.21%)	849,133 (6.74%)	773,057 (6.61%)	446,875 (3.68%)	450,812 (3.82%)	424,530 (3.57%)	409,762 (3.46%)
Unmapped reads	1,157,120 (9.77%)	1,178,203 (9.45%)	1,299,935 (10.31%)	1,264,607 (10.81%)	1,835,944 (15.13%)	1,704,246 (14.42%)	1,612,397 (13.55%)	1,666,199 (14.06%)

**FIGURE 1:**
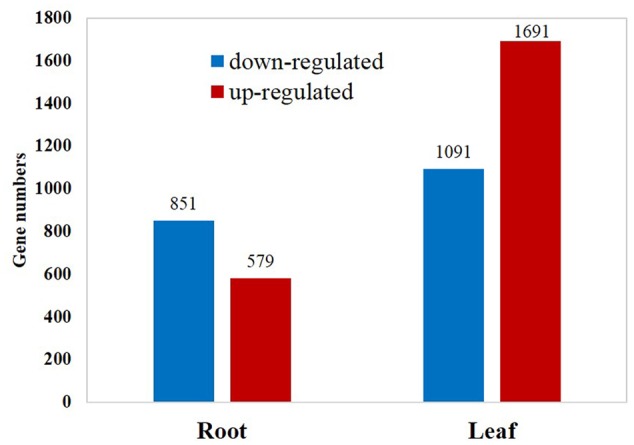
**Numbers of differentially expressed genes in response to cold treatment**.

**FIGURE 2:**
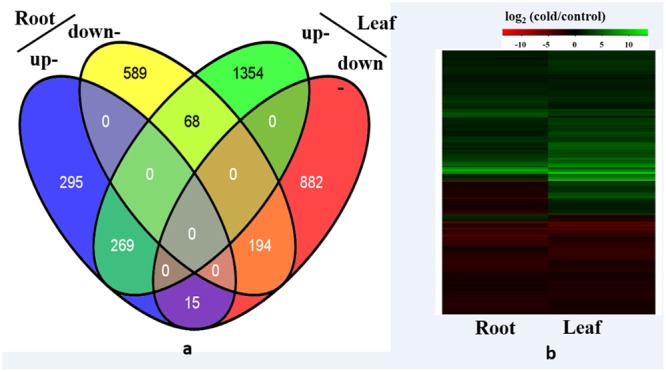
**Differentially expressed genes analysis. (a)** Venn diagram demonstrated the common and specific differentially expressed genes (DEGs) in roots and leaves, **(b)** Heat map demonstrated the expression profile DEGs in roots and leaves.

Among the 29,284 genes deposited in *Thellungiella* genome database, 22,414 (76.5%) genes were detected in the control and cold-treated libraries. A total of 2,782 and 1,430 differentially expressed genes (DEGs) were identified from leaves and roots, respectively (**Figure 1** and Supplementary Tables S1, S2). Under cold treatment, 579 and 1,691 genes were up-regulated, 851 and 1,091 genes were down-regulated in roots and leaves, respectively (**Figure 1**). Our results demonstrated that the expression patterns of the majority of DEGs were different in roots and leaves. For example, among the 1,691 up-regulated genes in leaves, only 269 (15.9%) were also induced in roots (**Figure 2**). Interestingly, some genes showed the opposite expression trend in roots and leaves upon cold treatment. For example, fifteen DEGs were up-regulated in root, but down-regulated in leaves. There were 68 DEGs down-regulated in root, but up-regulated in leaves (**Figure 2**). Moreover, 463 DEGs were with the same expression trend in roots and leaves, including 269 up-regulated and 194 down-regulated DEGs (**Figure 2** and Supplementary Table S1).

### Functional Analysis of DEGs

**FIGURE 3:**
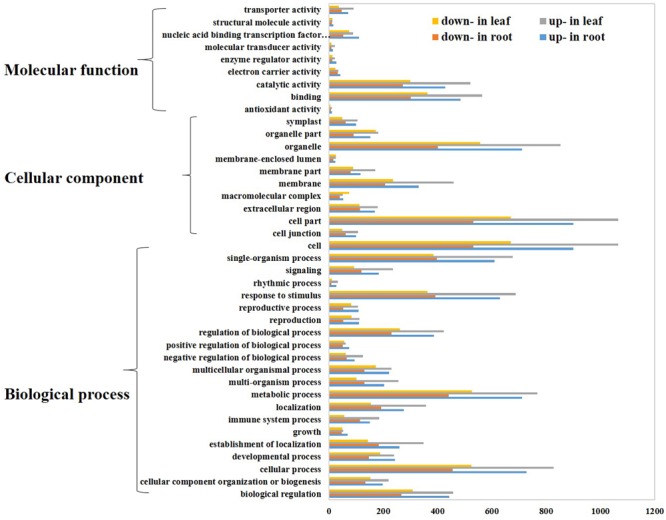
**Gene Ontology (GO) analysis of differentially expressed genes.** Genes were classified into three main categories: biological process, cellular component, and molecular function. The x-axis indicates the number of genes in a category, and the y-axis means the GO terms.

The DEGs were characterized by the assignment of gene ontology (GO) terms using Blast2GO (Conesa et al., 2005) program. GO enrichment analysis showed 241 and 374 GO terms were significant enriched in root and leaf, respectively (Supplementary Table S3). Then, we employed WEGO web-based tool to visualize the biological process, molecular function and cellular component main categories (**Figure 3**). In the cellular component category, the terms of “cell”, “cell part”, “organelle”, and “membrane” were enriched, implying the potential contribution of cell and cell structure in the process of *Thellungiella* response to cold condition. For the category of molecular function, “binding”, “catalytic activity” and “nucleic acid binding transcription factor activity” were the top terms. The most abundant terms of biological process were “cellular process”, “metabolic process”, “response to stimulus” and “single-organism process”, suggesting a high degree of metabolic activity changes upon cold treatment (**Figure 3**).

In order to obtain more biological information for understanding the molecular mechanism and regulatory network of *Thellungiella* cold tolerance, KEGG enrichment pathway analysis was performed. By applying a cut-off criterion of *Q*-value < 0.05 and *P*-value < 1E^-05^, the result showed that ten and fifteen pathways were significantly enriched from roots and leaves, respectively (**Table 2**). Previous studies demonstrated that many metabolic changes for enhancing freeze tolerance in *Arabidopsis*, such as increasing accumulation of soluble sugars and other compatible osmolytes (Wanner and Junttila, 1999). We found six pathways, “biosynthesis of secondary metabolites”, “metabolic pathways”, “nitrogen metabolism”, “tryptophan metabolism”, “cysteine and methionine metabolism”, and “sulfur metabolism” were enriched both in roots and leaves in response to cold. Interestingly, all these pathways were involved in a particular metabolic process, suggesting that the metabolic process was activated via cold treatment (**Table 2**). Proline is one of the most effective organic osmolytes in plants, and there is a positive correlation between proline accumulation and plant stress tolerance. A number of studies showed that proline played beneficial roles in plants when exposed to cold condition (Hayat et al., 2012). Previous studies demonstrated that *Thellungiella* contained higher levels of proline than *Arabidopsis* under non-stressed condition (Kant et al., 2006). Twenty-six genes involved in “Arginine and proline metabolism” pathway were found to be regulated by cold in our study. Most of these DEGs (18 of 26) were up-regulated, suggesting that these cold induced genes might promote the accumulation of proline in *Thellungiella* to enhance cold tolerance.

**Table 2:** The top enriched pathways of DEGs in roots and leaves.

Pathway	DEGs (%)	All genes	*P*-value	*Q*-value	Pathway ID
**Root: 1043 DEGs with KEGG pathway annotation**					
Biosynthesis of secondary metabolites	217 (20.81%)	1727 (11.11%)	2.99E-21	3.52E-19	ko01110
Metabolic pathways	326 (31.26%)	3484 (22.41%)	4.96E-12	2.93E-10	ko01100
Nitrogen metabolism	22 (2.11%)	76 (0.49%)	2.73E-09	1.07E-07	ko00910
Phenylpropanoid biosynthesis	49 (4.70%)	306 (1.97%)	1.04E-08	3.08E-07	ko00940
Flavonoid biosynthesis	33 (3.16%)	172 (1.11%)	3.47E-08	8.20E-07	ko00941
Phenylalanine metabolism	28 (2.68%)	151 (0.97%)	7.72E-07	1.52E-05	ko00360
Tryptophan metabolism	22 (2.11%)	127 (0.82%)	3.48E-05	5.87E-04	ko00380
Cysteine and methionine metabolism	21 (2.01%)	123 (0.79%)	6.48E-05	8.08E-04	ko00270
Sulfur metabolism	13 (1.25%)	56 (0.36%)	6.51E-05	8.08E-04	ko00920
Galactose metabolism	16 (1.53%)	80 (0.51%)	6.85E-05	8.08E-04	ko00052
**Leaf: 2419 DEGs with KEGG pathway annotation**					
Biosynthesis of secondary metabolites	408 (16.87%)	1727 (11.11%)	7.46E-21	9.55E-19	ko01110
Metabolic pathways	687 (28.40%)	3484 (22.41%)	3.31E-14	2.12E-12	ko01100
Photosynthesis - antenna proteins	16 (0.66%)	27 (0.17%)	2.62E-07	1.12E-05	ko00196
Nitrogen metabolism	29 (1.20%)	76 (0.49%)	1.40E-06	4.47E-05	ko00910
Photosynthesis	30 (1.24%)	81 (0.52%)	1.92E-06	4.92E-05	ko00195
Pyruvate metabolism	40 (1.65%)	125 (0.80%)	3.22E-06	6.87E-05	ko00620
Biosynthesis of unsaturated fatty acids	22 (0.91%)	53 (0.34%)	5.16E-06	9.44E-05	ko01040
Valine, leucine and isoleucine biosynthesis	17 (0.70%)	37 (0.24%)	1.20E-05	1.92E-04	ko00290
Sulfur metabolism	22 (0.91%)	56 (0.36%)	1.49E-05	2.12E-04	ko00920
Amino sugar and nucleotide sugar metabolism	47 (1.94%)	167 (1.07%)	2.30E-05	2.94E-04	ko00520
Glyoxylate and dicarboxylate metabolism	26 (1.07%)	74 (0.48%)	2.70E-05	3.14E-04	ko00630
Cysteine and methionine metabolism	37 (1.53%)	123 (0.79%)	3.48E-05	3.71E-04	ko00270
Circadian rhythm - plant	45 (1.86%)	162 (1.04%)	4.81E-05	4.74E-04	ko04712
Tryptophan metabolism	37 (1.53%)	127 (0.82%)	7.41E-05	6.44E-04	ko00380
Glycine, serine and threonine metabolism	29 (1.20%)	91 (0.59%)	7.55E-05	6.44E-04	ko00260

The “biosynthesis of unsaturated fatty acids” pathway was also enriched in *Thellungiella* leaves. A total of 22 genes involved in this pathway were affected by low temperature (**Table 2**). Previous study suggested that membrane lipid composition, especially the concentration of unsaturated fatty acid, is highly correlated with plant freezing tolerance (Thomas et al., 2012). The expression regulation of genes for unsaturated fatty acid synthesis might be a key factor contributing to cold tolerance in *Thellungiella*.

We observed that the pathways related to biosynthesis of phenylpropanoid and flavonoid were enriched in roots of *Thellungiella*. In addition, “Photosynthesis - antenna proteins” and “Photosynthesis” were enriched. Most of the DEGs in “Photosynthesis-antenna proteins” and “Photosynthesis” pathways were down-regulated in leaves, suggesting the adverse effect of low temperature on photosynthetic carbohydrate metabolism and photochemical reaction (Supplementary Table S2).

In *Arabidopsis*, environmental temperature affected the expression of clock component related genes, such as timing of cab expression 1 (*TOC1*), GIGANTEA (*GI*), circadian clock associated 1 (*CCA1*), and late elongated hypocotyl (*LHY*). Here, our results demonstrated that the pathway of “circadian rhythm - plant” was enriched in *Thellungiella* after cold treatment. The expression of many genes involved in circadian rhythm including *LHY, CCA1, TOC1* and *GI* were also induced or inhibited in *Thellungiella* upon cold treatment (**Table 2** and Supplementary Table S2).

### Validation of RNA-Seq Results Using Quantitative Real-Time PCR

**FIGURE 4:**
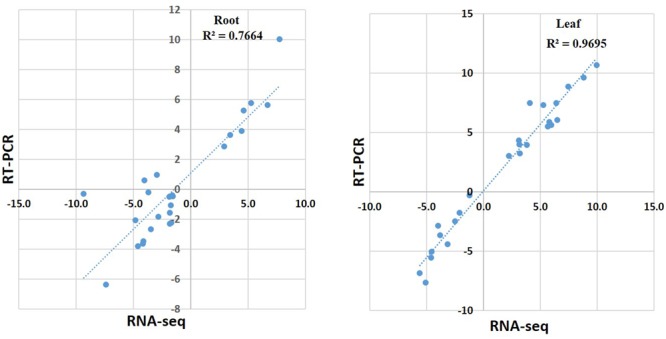
**Pearson’s correlation of RNA-seq and qRT-PCR results.** qRT-PCR validation of DEGs in roots and leaves under cold condition. The correlation of the fold change analyzed by RNA-Seq (*x*-axis) with data obtained using qRT-PCR (*y*-axis).

**FIGURE 5:**
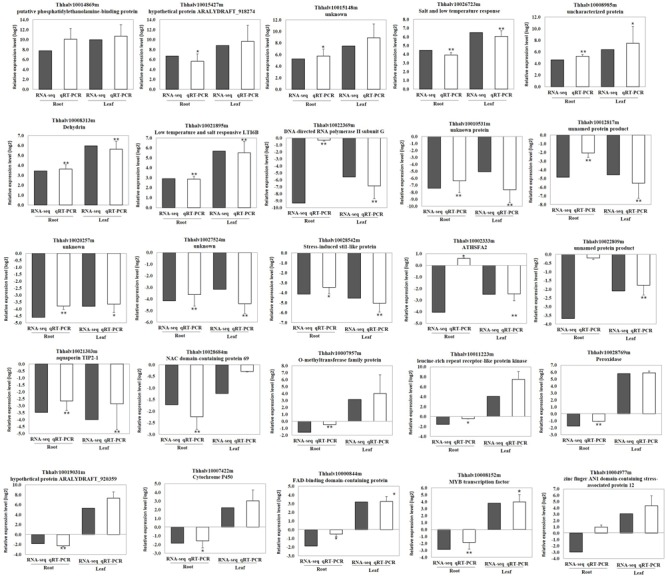
**Validation of gene expression by qRT-PCR.** Error bars indicate ± SE obtained from three biological repeats. Student’s *t*-test was performed to analyze the changes in the gene expression after treated with cold. ^∗∗^denotes the *p*-value <0.01 and ^∗^denotes the *p*-value <0.05.

To validate the RNA-seq data, quantitative real-time PCR (qRT-PCR) was performed for 25 genes with different expression levels and functional assignments (Supplementary Table S4). Among them, seven genes were significantly induced upon cold treatment both in roots and leaves. Two of these genes encoded salt and low temperature response protein, one encoded dehydrin. A putative phosphatidylethanolamine-binding protein gene, and three genes with unknown function were also selected for qRT-PCR validation. Other ten selected genes were down-regulated both in roots and leaves. These genes included one aquaporin *TIP2-1*, one NAC domain protein, one stress-induced sti1-like protein coding genes and several genes with unknown function. In addition, eight genes that down-regulated in roots but up-regulated in leaves were also selected for qRT-PCR analysis. These genes included O-methyltransferase family protein, peroxidase, cytochrome P450, leucine-rich repeat receptor-like protein kinase, MYB and zine finger AN1 domain-contain protein coding genes (Supplementary Table S4). The qRT-PCR results of these 25 genes in leaves were in agreement with the RNA-seq data. The relative expression level (log_2_ cold/control) estimated by RNA-seq and qRT-PCR were strongly correlated (*R*^2^=0.9695) in leaves (Supplementary Table S4 and **Figures 4, 5**). In roots, the majority of genes (23 of 25) showed similar expression patterns except Thhalv10004977m and Thhalv10002333m, which were down-regulated in RNA-seq, but up-regulated in qRT-PCR. In roots, the Pearson’s coefficient was 0.7664 which was lower than that in leaves (**Figure 4** and **Supplementary Figure S2**).

### Cold Related Transcription Factors

Studies showed that transcription factors played important roles in plant response to low temperature and other adverse stresses (Singh et al., 2002; Chen and Zhu, 2004; Agarwal P.K. et al., 2006). We observed that many transcript factors were response to cold in *Thellungiella*. Among the 1,430 DEGs in roots, 159 genes (11.1%) were annotated as different types of transcription factors (**Table 3**). About half of these transcription factor genes (74 of 159) were up-regulated, the rest 85 genes were down-regulated. According to functional annotation, these transcription factors were classified into 18 categories, such as abscisic acid responsive, NAC, Zinc finger domain, AP2, MYB, bHLH and WRKY etc. The transcription factors Zinc finger domains (27 genes, 17.0%), ethylene responsive (11 genes, 6.9%) and calcium ion binding (10 genes, 6.3%) were the three major families of the cold-regulated transcription factors in roots.

**Table 3:** Cold-regulated transcription factors.

Category	Number of transcription factors in root			Number of transcription factors in leaf		
	Total	Up-regulated	Down-regulated	Total	Up-regulated	Down-regulated
Abscisic acid responsive	4	3	1	1	1	0
AP2	1	0	1	2	1	1
Auxin-responsive	4	2	2	5	2	3
Basic leucine zipper	3	1	2	1	0	1
bHLH	4	0	4	4	1	3
BTB and TAZ domain	1	1	0	1	0	1
Calcium ion binding	10	0	10	6	5	1
Ethylene responsive	11	4	7	5	3	2
F-box	5	1	4	5	0	5
Heat shock protein	1	0	1	9	5	4
Homeobox-leucine zipper	1	1	0	4	0	4
Leucine-rich repeat	6	1	5	7	2	5
MADS-box	2	2	0	1	0	1
MYB	6	3	3	10	3	7
NAC-domain	2	1	1	9	7	2
WRKY	3	0	3	12	11	1
Zinc finger domain	27	15	12	43	33	10
Ulassification	68	39	29	107	57	50

**Total**	**159**	**74**	**85**	**232**	**131**	**101**

Among the 2,782 DEGs in leaves, 232 (8.3%) were transcription factors, including 131 up-regulated and 101 down-regulated genes. These transcription factors were classified into 18 categories (**Table 3**). Among these differential expressed transcription factors in leaves, Zinc finger domain, WRKY and MYB are enriched. Heat shock proteins (HSPs) were implicated in plant heat stress tolerance (Nover et al., 2002). Here, we found five HSPs were up-regulated and four HSPs were down-regulated upon cold treatment. WRKY transcription factors played important roles in plant responses to biotic and abiotic stress (Eulgem et al., 2000). Twelve differentially expressed WRKY transcription factor genes were identified in leaves, and eleven of them were up-regulated. NAC transcription factors were involved in many aspects of plant growth and development, and response to abiotic stress (Nuruzzaman et al., 2013). In our RNA-seq data, nine and three differentially expressed NAC transcription factor genes were identified in leaves and roots, respectively. These results indicated that HSF, WRKY and NAC transcription factors were involved in plant responses to various stresses, and suggested that cold stress might share common molecular mechanism with other abiotic stresses. In addition, we noticed that the expression of ten calcium ion binding transcription factors was all suppressed in roots upon cold treatment, while most of them were induced in leaves, implying that these genes might be functioning in different ways in roots and leaves (**Table 3** and Supplementary Table S1). These results suggested the existence of differences in cold responsive regulatory networks between roots and leaves in *Thellungiella*.

Previous studies showed that some MYB transcription factors were involved in cold stress tolerance, such as *AtMYB15* in *Arabidopsis* (Agarwal M. et al., 2006), *MYB55* and *OsMYBS3* in rice (Su et al., 2010; Elkereamy et al., 2012). In our data, the expression of six and ten *MYB* genes was altered after cold treatment in roots and leaves, respectively (**Table 3** and Supplementary Tables S1, S2). Interestingly, Thhalv10008152m encoding a MYB transcription factor displayed opposite expression trend in roots and leaves. In roots, the expression of this gene was inhibited by cold treatment, while it was induced in leaves (**Figure 5**).

Basic helix-loop-helix (*bHLH*)-type transcription factors played important regulatory roles in diverse biological processes in plants (Toledoortiz et al., 2003). The latest evidences showed that *bHLH* transcription factor acted as positive regulators of CBF-pathway and conferred cold tolerance in plants (Feng et al., 2012; Peng et al., 2013; Xu et al., 2014). We found that the expression of four and four *bHLH* genes was altered after cold treatment in *Thellungiella* roots and leaves, respectively (**Table 3** and Supplementary Tables S1, S2). However, only one of these *bHLH* gene (Thhalv10025656m) was induced by cold, and the others were down-regulated in response to cold treatment. Functional annotation showed that Thhalv10025656m was homologous of *bHLH69* of *Arabidopsis*, and named as *TsbHLH69*. In *Arabidopsis, bHLH69* contributed to the regulation of circadian periodicity by reducing the expression of *LHY* and *TOC1* (Hanano et al., 2008; Harmer, 2009). These data suggested that the cold-induced *TsbHLH69* might participate in the regulation of rhythm of *Thellungiella* under cold condition.

### Cold Regulation of Genes Related to Plant Hormone Biosynthesis and Signaling

In this study, we found that the expression of many genes related to plant hormone biosynthesis and signaling were altered upon cold treatment (Supplementary Tables S1, S2). A total of 134 DEGs were related to plant hormone biosynthesis and signaling in leaves, 71 of which were up-regulated and 73 of which were down-regulated. DELLA, an key factor in gibberellins (GA) signaling, was also involved in the signal transduction of other hormones suggesting that DELLA functions as a modulator of plant development and response to stresses (Achard et al., 2003; Willige et al., 2007). Studies showed that DELLA contributed to CBF1-induced cold acclimation and was considered as components of CBF1-mediated cold stress response (Achard et al., 2008). We found the expression of a DELLA encoding gene (Thhalv10015535m) was slightly induced in both roots (1.40 fold) and leaves (1.39 fold) upon cold treatment. Meanwhile, gibberellin 2-oxidase (Thhalv10008179m), an enzyme inactivating the bioactive gibberellins (GAs), was significantly induced (2.6-fold) (Supplementary Table S2). These results implied that GA metabolism and signaling might contribute to cold stress tolerance in *Thellungiella*.

### Other Cold-Regulated (*COR*) Genes

Studies demonstrated that the expression of *COR* genes was strongly induced after plants were shifted to cold temperature (Hajela et al., 1990; Griffith et al., 2007). RNA-seq results revealed five *COR* genes including *COR27, COR47*, and three *COR15* were dramatically induced in both roots and leaves under cold treatment (**Table 4**). Interestingly, we found that the expression levels of all three *COR15* increased by 1,000-fold in leaves after cold treatment. Among all these *COR* genes, *COR15* represented the most induced genes, whose expression level increased more than 8,000-fold to compare with the control. Sequence alignment results showed that the DNA sequences of these three *COR15* genes were highly similar, implying these genes might have the same functions in cold tolerance (**Supplementary Figure S1**). The *COR47* gene encoding a member of dehydrin has been isolated in several plant species. Previous studies showed that the promoter region of *COR47* contained C-repeat, dehydration-responsive element, low temperature-responsive element (CRT/DRE/LTREs) and ABA regulatory element (ABRE). *COR47* was the downstream gene of *CBF/DREB1*, and *CBF/DREB1* binds to the promoter of *COR47* to induce its expression (Jaglo-Ottosen et al., 1998). Functional annotation and sequence alignment showed that Thhalv10027405m, a *TsCBF/DREB1* gene, was a homolog of *Arabidopsis CBF1/2/3*. RNA-seq data revealed that Thhalv10027405m was up-regulated in both roots and leaves under cold treatment (**Table 4** and Supplementary Table S2). The expression profiles of *TsCBF/DREB1* and *COR47* in *Thellungiella* was consistent with their counterparts in *Arabidopsis*, suggesting that *CBF/DREB1* and *COR47* genes might play important roles in *DREB1/CBF*-dependent cold signaling pathway in *Thellungiella.*

**Table 4:** The expression profile of cold-regulated genes.

Gene	Annotation	Relative expression level (cold/control)				Expression trend
		log2 (leaf)	*Q*-value (leaf)	log2 (root)	*Q*-value (root)	
Thhalv10017395m	Cold response protein (*COR15*)	13.40	1.000	3.36	0.830	Up
Thhalv10017393m	Cold response protein (*COR15*)	10.73	0.961	7.44	0.540	Up
Thhalv10017394m	Cold response protein (*COR15*)	10.22	0.943	8.11	0.932	Up
Thhalv10003264m	*COR27*	9.26	0.979	1.18	0.809	Up
Thhalv10008706m	*COR47*	6.80	0.999	6.54	0.998	Up
Thhalv10027405m	*CBF/DREB1*	2.85	0.89	1.61	0.90	Up

In addition, our RNA-seq data indicated that there might be more genes involved in *DREB1/CBF* pathway in *Thellungiella.* For example, we found *DREB2B* gene (Thhalv10021161m) was up-regulated in leaves under cold treatment (Supplementary Table S2). In addition to *COR47* gene, we observed other two dehydrin genes, Thhalv10010821m and Thhalv10008313m were also induced by cold treatment (**Figure 5** and Supplementary Table S2).

In *Arabidopsis*, INDUCER OF CBF EXPRESSION 1 (*ICE1*) could induce *CBF3* expression by binding to its promoter. It was an important upstream regulatory factor in *DREB1/CBF* signaling pathway (Chinnusamy et al., 2003). In *Thellungiella*, we observed that the expression of *ICE1* (Thhalv10004059m) were up-regulated in roots upon cold treatment (1.57-fold). However, the expression level of *ICE1* was similar in cold treated and control samples in leaves. These results indicated that the function of *ICE1* might be different in *Arabidopsis* and *Thellungiella.*

Aquaporins (or water channel proteins) played a crucial role in plant water relations. According to their distinct sub-cellular localization, aquaporins could be divided into four subgroups, the tonoplast intrinsic proteins (TIPs), plasma membrane intrinsic proteins (PIPs), Nod26-like intrinsic membrane proteins (NIPs) and small basic intrinsic proteins (SIPs). TIPs were abundantly expressed in vacuole (Maurel, 2007) and played an important role in maintenance of the intracellular space by controlling the water influx in vacuole (Leitão et al., 2014). A member of tonoplast intrinsic proteins *GhTIP1;1* was responsive to cold stress and contributed to freezing-tolerance in cotton (Li D.D. et al., 2009). In the current study, five cold regulated aquaporin genes including three *TIPs*, one *PIPs* and one *NIPs* were identified. RNA-seq and qRT-PCR results demonstrated that *TIP2-1* (Thhalv10021303m) was significantly down-regulated in both roots and leaves when the plants were under cold treatment. Another two *TIP* genes, Thhalv10011730m (also annotated as *TIP2-1*) and Thhalv10002084m (*TIP4-1*), were also significantly down-regulated upon cold treatment. These results suggested that the decreased expression of *TIP* genes might be beneficial to reduce water in/out of vacuole which is important for maintaining the stability of the cells in cold condition (**Figure 5** and Supplementary Tables S1, S2). Moreover, we observed that the expression of *PIP2-7* (Thhalv10025940m) was significantly decreased, suggesting that the water in/out of the cell might be reduced.

## Conclusion

**FIGURE 6:**
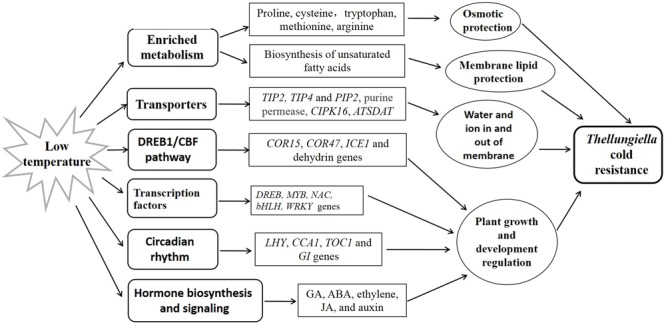
**Simple model deduced based on the transcriptome data**.

In this study, we compared the transcriptome of *Thellungiella* roots and leaves in response to cold treatment using RNA-seq. We identified a number of cold-responsive genes which were involved in different pathways closely related to environmental adaptation and other biological processes, suggesting the complex responses of *Thellungiella* toward cold stress (**Figure 6**). Our findings provided an overall picture of the regulatory network in response to cold stress in *Thellungiella*. These cold-responsive genes could be targeted as potential candidates for further functional validation, and have potential application value for increasing cold tolerance in crops.

## Materials and Methods

### Plant Materials

Seeds of *T. salsuginea* (Shandong ecotype) were surface-sterilized and plated on 1/2 MS-agar plates for synchronize germination at 4°C for a week. The plates were moved to growth chamber with 16 h light at 26°C with light intensity of 3000 lx and 8 h dark at 22°C. Seven-day-old seedlings were transferred to soil for 5 weeks. For cold treatment, seedlings were exposed to a growth chamber under a 16/8 h light/dark regime at 8/4°C for 24 h. For the control, the seedlings were grown at 22/16°C. Leaves and roots of control and cold treated seedlings were collected, frozen immediately in liquid nitrogen, and stored at -80°C. To minimize the plant to plant variation, nine individuals were used as one biological replicate, the tissue from nine individuals were pooled into one independent biological replicate. For both leaves and roots, three biological replicates were prepared.

### RNA Isolation, cDNA Library Construction, and Sequencing

Total RNA was isolated from leaves and roots using RNAiso Reagent (Takara, China), and treated with DNase I (Takara, China) to remove the contaminated genomic DNA according to the manufacturer’s protocols. RNA quality was detected by electrophoresis on 1.0% agarose gels and NanoDrop. The mRNA was enriched and cleaved into short fragments (about 200 nt). The mRNA fragments were used as templates to synthesize the first strand cDNA using random hexamer-primer. The first strand cDNA was further incubated with DNA polymerase I, buffer, dNTPs and RNase H to synthesize the second strand. Following end repair, a single nucleotide (adenine) was added, and then sequencing adaptors were ligated to the fragments. Finally, the fragments were purified and enriched with PCR amplification to construct cDNA library. Two biological replicates of each sample were used for RNA-Seq via Illumina HiSeq^TM^2000 platform by Beijing Genomics Institute (BGI).

### Bioinformatics Analysis of RNA-Seq Data

To acquire clean reads, the low-quality reads, adaptor sequences, and empty reads were removed. All clean reads were mapped with the genome sequences of *T. salsuginea*^2^ using SOAP2 program under the criterion of no more than two mismatches in the alignment (Li R. et al., 2009). The gene expression level was calculated using RPKM (Reads per Kb per Million reads) method according to previous studies (Morrissy et al., 2009; Qu et al., 2016). The relative gene expression level between different samples was calculated by log_2_ ratio. Differentially expressed genes (DEGs) were identified using NOIseq under the criteria of probability ≥0.8 and the absolute value of log^2^Ratio ≥ 1. The probability (*P*-value) was calculated according to the manufacturer’s protocol with the default parameters (Tarazona et al., 2011).

Gene Ontology annotation was conducted using Blast2GO (Conesa et al., 2005) by comparing all DEGs with GO terms in the database, which covered three domains: cellular component, molecular function and biological process^3^. The significantly enriched GO terms in DEGs were identified using hypergeometric test comparing to the genome background under the standard of *p*-value ≤ 0.05. Then GO picture was generated using WEGO^4^ (Web Gene Ontology Annotation Plot) (Ye et al., 2006). KEGG (Kyoto Encyclopedia of Genes and Genomes) pathway analysis was performed by mapping the DEGs to specific biochemical pathways in KEGG database^5^. Significantly enriched metabolic pathways or signal transduction pathways were identified using enrichment analysis by comparing DEGs with the whole genome background.

### qRT-PCR Validation of RNA-Seq Results

The primers used for qRT-PCR validation were designed using primer premier 5.0 software^6^ and were listed in Supplementary Table S5. The primers were designed according to the transcript sequences of *T. salsuginea* downloaded from database ^7^. qRT-PCR was performed in ABI7500 Real Time System (Applied Biosystems) using SYBR Green I (Roche) with the following reaction: 94°C 10 min; 94°C 15 s, 60°C 10 s and 72°C 25 s for 40 cycles. All reactions were performed in biological triplicates, ubiquitin, and actin were used as internal reference genes. The relative expression of genes was calculated by the software of ABI7500 Real-Time PCR System using the 2^-△△ Ct^ method.

## Author Contributions

Manuscript draft: JW, CZ, and XW; Analyzing data: CZ and FC; Experiment: JW, QZ, LH, SZ, HX, JQ, TL, and YZ; Conception and supervision of the research: CZ and XW.

## Conflict of Interest Statement

The authors declare that the research was conducted in the absence of any commercial or financial relationships that could be construed as a potential conflict of interest.

## Footnotes

1 https://www.ncbi.nlm.nih.gov/sra/

2 http://www.phytozome.net/thellungiella.php

3 http://www.geneontology.org/

4 http://wego.genomics.org.cn/cgi-bin/wego/index.pl

5 http://www.genome.jp/kegg/

6 www.premierbiosoft.com

7 https://phytozome.jgi.doe.gov/pz/portal.html#info?alias=Org_Esalsugineum

## Supplementary Material

FIGURE S1 **Sequence alignment of three *COR15* genes**.

FIGURE S2 **Hierarchical cluster and correlation analysis between qRT-PCR and RNA-seq**.
