# Supplementary figures and images for: Genome-Wide Analysis of Gene Expression Provides New Insights into Cold Responses in Thellungiella salsuginea
Source: Front Plant Sci. 2017 May 8;8:713. doi: 10.3389/fpls.2017.00713 (PMC5420556; doi:10.3389/fpls.2017.00713)

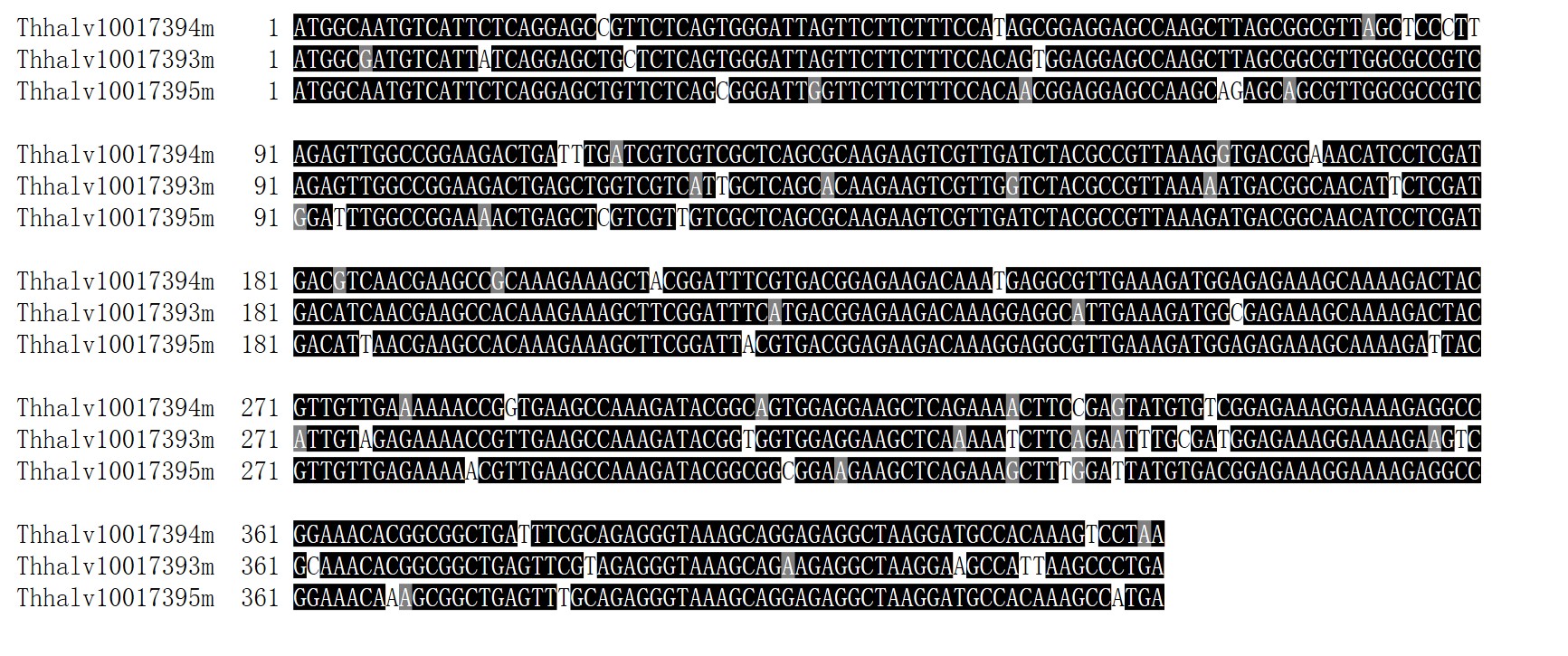

Supplement: FIGURE S1 — Sequence alignment of three COR15 genes. [file Image_1.JPEG]

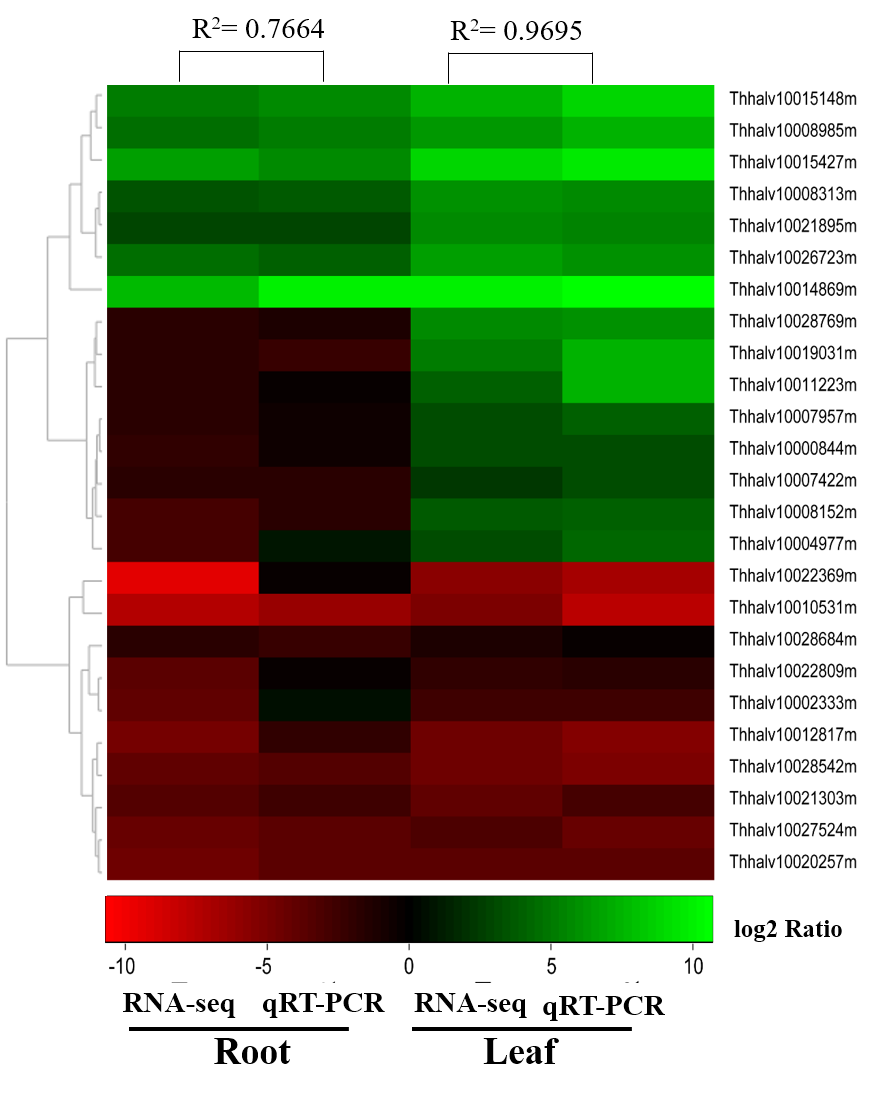

Supplement: FIGURE S2 — Hierarchical cluster and correlation analysis between qRT-PCR and RNA-seq. [file Image_2.PNG]
